# Supplementary material for: Evidence supported by Mendelian randomization: impact on inflammatory factors in knee osteoarthritis
Source: Front Med (Lausanne). 2024 May 28;11:1382836. doi: 10.3389/fmed.2024.1382836 (PMC11165061; doi:10.3389/fmed.2024.1382836)
Supplement: Supplementary file 1 [file Data_Sheet_1.ZIP › data/GCST90274778_5e6/778.leaveoneout.pdf]

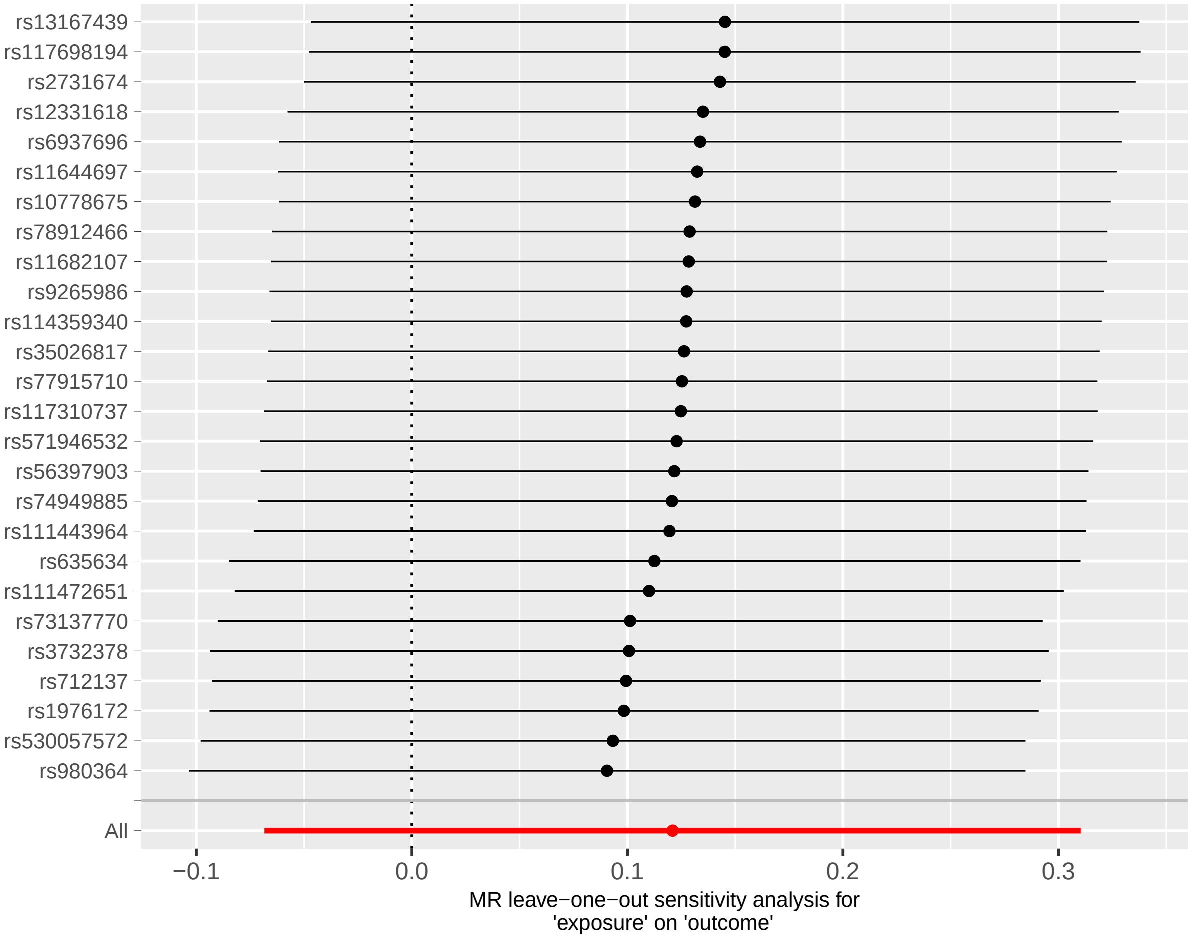
rs13167439
rs117698194
rs2731674
rs12331618
rs6937696
rs11644697
rs10778675
rs78912466
rs11682107
rs9265986
rs114359340
rs35026817
rs77915710
rs117310737
rs571946532
rs56397903
rs74949885
rs111443964
rs635634
rs111472651
rs73137770
rs3732378
rs712137
rs1976172
rs530057572
rs980364
All
−0.1
0.0
0.1
0.2
0.3
MR leave−one−out sensitivity analysis for
'exposure' on 'outcome'
